# Synthesis and Characterization of a NiCo_2_O_4_@NiCo_2_O_4_ Hierarchical Mesoporous Nanoflake Electrode for Supercapacitor Applications

**DOI:** 10.3390/nano10071292

**Published:** 2020-06-30

**Authors:** Xin Chen, Hui Li, Jianzhou Xu, F. Jaber, F. Musharavati, Erfan Zalnezhad, S. Bae, K.S. Hui, K.N. Hui, Junxing Liu

**Affiliations:** 1 School of Mechanical Engineering, Xinjiang University, Urumchi 830046, China; cx5585208@hotmail.com; 2 Department of Chemical Engineering, Hanyang University, 222 Wangsimni-ro, Seongdong-gu, Seoul 04763, Korea; hui.li.alan@gmail.com; 3 Department of Mechanical Engineering, Hanyang University, 222 Wangsimni-ro, Seongdong-gu, Seoul 04763, Korea; xujianzhoulvqingqing@gmail.com; 4 Department of Biomedical Engineering, Ajman University, Ajman 2758, UAE; f.jaber@ajman.ac.ae; 5 Department of Mechanical and Industrial Engineering, College of Engineering, Qatar University, P.O. Box 2713, Doha 2713, Qatar; farayi@qu.edu.qa; 6 Department of Chemical and Biomedical Engineering, University of Texas at San Antonio (UTSA), San Antonio, TX 78249, USA; 7 Department of Architectural Engineering, Hanyang University, Seoul 04763, Korea; liujx128119@hanyang.ac.kr; 8 School of Engineering, University of East Anglia, Norwich, NR4 7TJ, UK; k.hui@uea.ac.uk; 9 Institute of Applied Physics and Materials Engineering, University of Macau, Avenida da Universidade, Taipa, Macau; bizhui@umac.mo

**Keywords:** supercapacitors, electrodeposition, NiCo_2_O_4_, nanostructure

## Abstract

In this study, we synthesized binder-free NiCo_2_O_4_@NiCo_2_O_4_ nanostructured materials on nickel foam (NF) by combined hydrothermal and cyclic voltammetry deposition techniques followed by calcination at 350 °C to attain high-performance supercapacitors. The hierarchical porous NiCo_2_O_4_@NiCo_2_O_4_ structure, facilitating faster mass transport, exhibited good cycling stability of 83.6% after 5000 cycles and outstanding specific capacitance of 1398.73 F g^−1^ at the current density of 2 A·g^−1^, signifying its potential for energy storage applications. A solid-state supercapacitor was fabricated with the NiCo_2_O_4_@NiCo_2_O_4_ on NF as the positive electrode and the active carbon (AC) was deposited on NF as the negative electrode, delivering a high energy density of 46.46 Wh kg^−1^ at the power density of 269.77 W kg^−1^. This outstanding performance was attributed to its layered morphological characteristics. This study explored the potential application of cyclic voltammetry depositions in preparing binder-free NiCo_2_O_4_@NiCo_2_O_4_ materials with more uniform architecture for energy storage, in contrast to the traditional galvanostatic deposition methods.

## 1. Introduction

Owing to their long cycling life, faster charge–discharge processes, and high power density, supercapacitors are evolving as significant and reliable devices for energy storage [1,2,3]. Their performance is affected by the porosity, size, and morphology of the electrode materials. Significant attention has been concentrated on the study of different electrode materials. Because of the distinctive morphologies of carbons and their outstanding thermal, mechanical, and chemical stability, they have attracted much attention for numerous applications, such as sensing, catalysis, and energy conversion and storage [4]. Commonly, ordered nanoporous carbons, fullerenes, and carbon nanotubes have been extensively applied for environmental and energy applications [5]. Nevertheless, the intricate fabrication processes of fullerenes and carbon nanotubes make it difficult to develop their full potential in different fields [6,7].

Due to their significant electrochemical performance, transition metal oxides have been selected as electrode materials for supercapacitors. Compared with nickel oxide [8,9] and cobalt oxide [10,11,12], nickel cobaltite (NiCo_2_O_4_) is a widely chosen electrode material owing to its low cost, environmental benignity, stable structure, and rich electroactive sites [4,5,6,7]. However, NiCo_2_O_4_-based electrode materials [13,14,15] show inferior structural stability and electrical conductivity, requiring further improvement for potential high energy density applications [16,17,18,19,20]. These limitations can be tackled by precisely designing NiCo_2_O_4_-based metal oxide nanostructures with other recognized capacitive oxides to fulfill the requirements of high-performance supercapacitors.

Yedluri et al. [21] synthesized NiCo_2_O_4_/NiCo_2_O_4_ nanoflake arrays on NF using a hydrothermal technique that involved a long reaction time at a high temperature of 350 °C for 3 h. The NiCo_2_O_4_/NiCo_2_O_4_ nanocomposite had a high specific capacitance of 2312 F g^−1^ at a current density of 2 mA cm^−2^, as well as high cycling stability. In this present study, we fabricated the ultrathin NiCo_2_O_4_ nanoflakes through the traditional hydrothermal technique as the “first laminate”. Then, the “second laminate” of the NiCo_2_O_4_ nanoflakes was synthesized on the “first laminate” by cyclic voltammetry deposition. Compared to bare NiCo_2_O_4_ array electrodes, the laminated ones showed improvement in electrochemical performance, with higher capacitance and better cycling stability. The unique properties of laminated nanoflake arrays, including fast ion and electron transfer, good strain accommodation, and a large number of active sites, make this composite novel and useful for energy storage. The NiCo_2_O_4_@NiCo_2_O_4_ material exhibited good cycling stability of 83.6% after 5000 cycles and outstanding specific capacitance of 1398.73 F g^−1^ at the current density of 2 A g^−1^, which is higher than the core–shell NiCo_2_O_4_@NiCo_2_O_4_ structure of 1264 F g^−1^ and the NiCo_2_O_4_ structure of 556 F g^−1^ [19,20].

In recent years, with advances in technology in different fields (biomedical, sports, consumer electronics, environmental, clean energy, and mobility), wearable and flexible microelectronic systems and devices have become very important [18]. Supercapacitors (in solid state) are usually fabricated in a sandwich structure containing a polymer gel electrolyte amid two electrodes. Herein, a solid-state supercapacitor was fabricated, with the NiCo_2_O_4_@NiCo_2_O_4_ deposited on NF as the positive electrode and the active carbon (AC) deposited on NF as the negative electrode, delivering a high energy density of 46.46 Wh kg^−1^ at the power density of 269.77 W kg^−1^.

## 2. Experimental Details

### 2.1. Synthesis of NiCo_2_O_4_ Nanoflake Arrays on Nickel Foam

First, to eliminate oxides and impurities from nickel foam, the samples were etched in 5% hydrochloric acid, cleaned in ethanol, cleaned with distilled water, and dried in an oven. Second, the cleaned samples (1 × 1 cm^2^) were located on 100 mL of Teflon to build NiCo_2_O_4_ nanoflakes through the hydrothermal method. The hydrothermal solution was prepared by mixing and dissolving 15 mmol of urea, 6 mmol of NH_4_F, 2 mmol of cobalt nitrate hexahydrate, and 1 mmol of nickel nitrate hexahydrate in 70 mL of distilled water, which was magnetically stirred for 20 min at ambient temperature. The final product was transferred into the 120 mL autoclave. The autoclave was transferred into an oven and kept for 3 h at 120 °C, then cooled to ambient temperature afterward. The pink-colored samples (cobalt–nickel hydroxide coated nickel foam) were cleaned with distilled water numerous times and dried in air.

### 2.2. Synthesis of NiCo_2_O_4_@ NiCo_2_O_4_ Electrode

NiCo_2_O_4_ with laminated structure was electrochemically deposited onto NiCo_2_O_4_ nanoflake array coated nickel substrates. A conventional three-electrode cell at ambient temperature was used for electrodeposition. Pt foil, NiCo_2_O_4_-coated NF, and saturated Ag/AgCl were used as the counter, working, and reference electrodes, respectively. Co_2*x*_Ni*_x_*(OH)_2_, the electrodeposition electrolyte, was made of 70 mL of a 0.1 M metal ion solution, with a Co^2+^/Ni^2+^ concentration ratio of 2:1. NiCo_2_O_4_ nanoflake arrays were coated by Co*_x_*Ni_1−*x*_(OH)_2_ acicular flakes with a potential of −1.2–0.5V for 30 s. Dynamic potential cyclic scanning for electrodeposition was used for electrodeposition. The sequence and range of potential scanning was 0→−1.2→0.5→0 V. Samples were obtained by deposition for different durations, including 20 s, 30 s, 40 s, and 50 s. Cyclic voltammetry (CV) test indicated that the sample electrodeposited for 30 s showed the best electrochemical performance.

After electrodeposition, to eliminate the residues from the samples, they were cleaned and washed in an ultrasonic bath with ethanol and double deionized water and dried at ambient temperature. The samples were annealed for 2 h at 350 °C to convert Co*_x_*Ni_1−*x*_(OH)_2_ to NiCo_2_O_4_ on the first laminate NiCo_2_O_4_ nanoflake arrays.

### 2.3. Characterizations

The crystalline structure and phase purity of the products were identified by X-ray diffraction (XRD) using a D8 Advance automated X-ray diffractometer system with Cu-Kα (λ = 1.5418 Å) radiation at 40 kV and 40 mA, ranging from 5° to 90° at room temperature. Transmission electron microscopy (TEM, JEM-2100F (JEOL, Seoul, South Korea, at 200 kV acceleration voltage), selected area electron diffraction (SAED, Seoul, South Korea), field emission scanning electron microscopy (FESEM, LEO-1550, at 5 kV applied voltage, Seoul, South Korea), and energy-dispersive X-ray spectroscopy (EDS, Seoul, South Korea) were used to characterize the structure and morphologies of the samples.

### 2.4. Electrochemical Characterizations

A three-electrode electrochemical RST 5100F workstation was used to electrochemically characterize the samples in 2 M KOH. NiCo_2_O_4_@NiCo_2_O_4_, Hg/HgO, and platinum foil were the working, reference, and counter electrodes, respectively. Electrochemical impedance spectroscopy (EIS) tests were conducted in the 0.01–100 kHz frequency range, with a potential amplitude of 5 mV. The CV test was carried out in a 0–0.6 V potential window at 5, 10, 30, and 50 mV s^−1^. Galvanostatic charge–discharge experiments were performed in a 0–0.55 V potential range at 2, 4, 6, 8, 10, and 20 A g^−1^.

The following equation was used to calculate the specific capacitance (*C*):

(1) $$C=\frac{I\Delta t}{M\Delta V}$$

where *M*, Δ*t*, Δ*V*, and *I* are the active material’s mass, total discharge time, voltage drop through the discharge process, and the discharge current, respectively.

## 3. Results and Discussion

**Figure 1:**
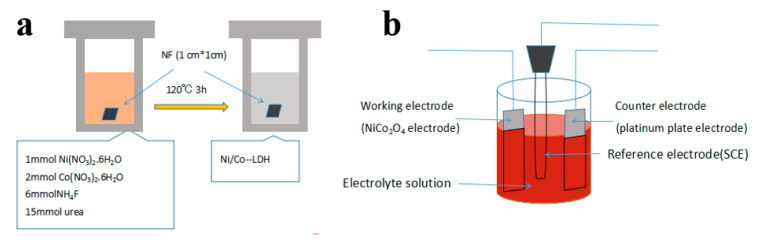
Schematic of the preparation of Co*_x_*Ni_1−*x*_(OH)_2_ nanoflake arrays on Ni foam by (**a**) hydrothermal and (**b**) electrodeposition methods.

Figure 1a,b illustrate the schematic of nickel–cobalt-layered double hydroxide synthesized through hydrothermal and electrodeposition methods. The hydrothermal reaction involves four parts [21,22], as follows:

H_2_NCONH_2_+H_2_O → 2NH_3_+CO_2_ (2)

NH_3_·H_2_O → NH_4_^+^+OH^−^ (3)

*x*Co^2+^+(1−*x*)Ni^2+^+6NH_3_ ↔ [CoxNi_(1−*x*)_(NH_3_)_6_]^2+^ (4)

[Co*_x_*Ni_(1−*x*)_(NH_3_)_6_]^2+^+2OH^−^ → Co*_y_*Ni_(1−*y*)_(OH)_2_+6NH_3_ (5)

The electrodeposition and metal hydroxide precipitation reactions are as follows [23]:

NO^3−^ +7H_2_O +8e^−^ → NH_4_^+^+10HO^−^ (6)

*x*Ni^2+^ + 2*x*Co^2+^ + 6*x*HO^−^ → Ni*_x_*Co_2*x*_(OH)_6*x*_ (7)

Metal hydroxide converts to NiCo_2_O_4_ according to the following reaction [4]:

Ni*_x_*Co_2*x*_(OH)_6*x*_ + *x*O_2_/2 → *x*NiCo_2_O_4_ + 3*x*H_2_O (8)

**Figure 2:**
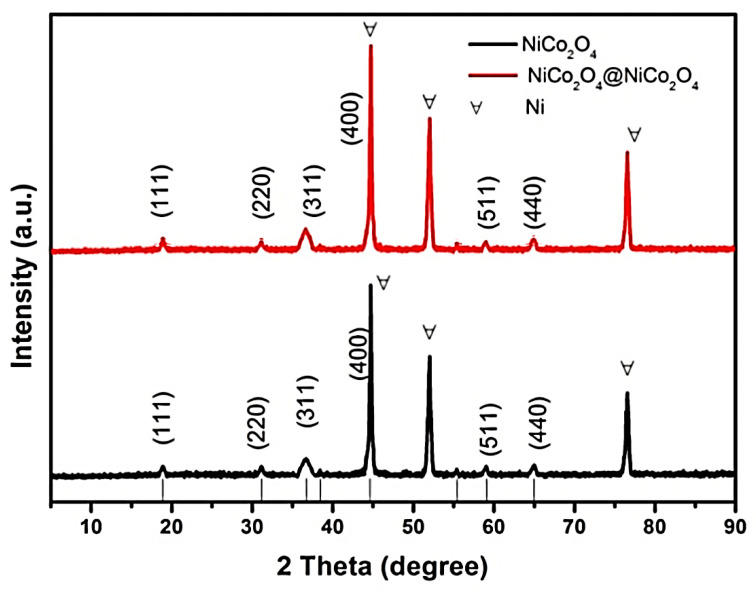
XRD analysis of NiCo_2_O_4_@NiCo_2_O_4_ nanoflake and bare NiCo_2_O_4_ electrodes.

Figure 2 shows the XRD phase analysis of NiCo_2_O_4_@NiCo_2_O_4_. There are several distinct diffraction peaks belong to the cubic spinel NiCo_2_O_4_ structure (JCPDS card no. 20-0781) [24]. The diffraction peaks at 44.7°, 52.1°, and 76.5° belong to nickel foam. The peaks at 18.906°, 31.148°, 36.696°, 44.622°, 59.094°, and 64.980° are respectively identified as the (111), (220), (311), (400), (511), and (440) planes belonging to the NiCo_2_O_4_.

**Figure 3:**
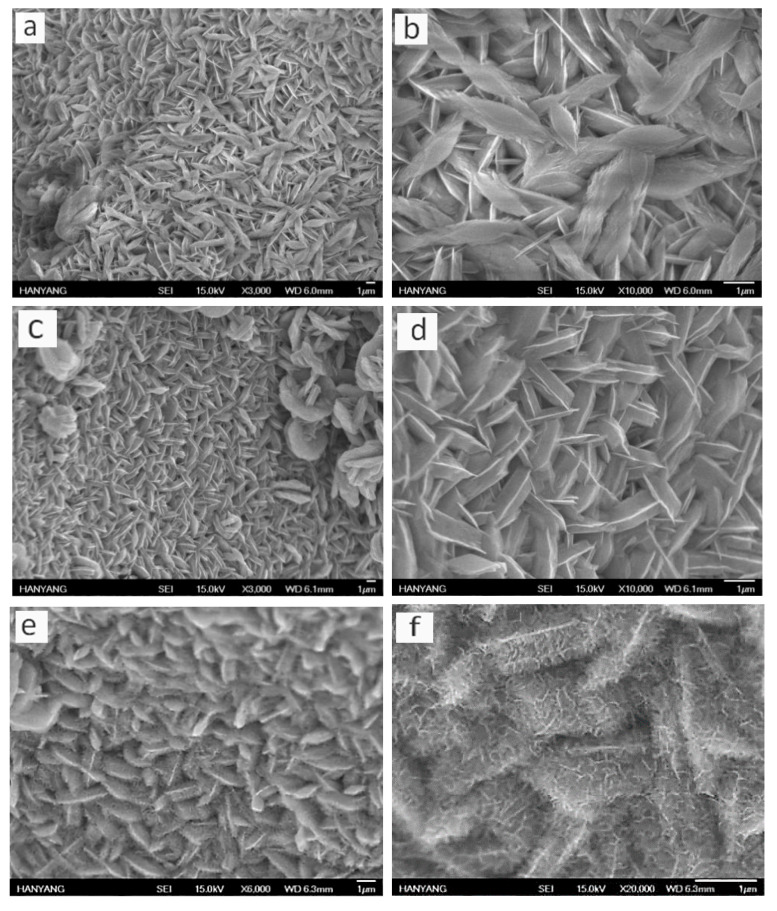
Scanning electron microscope (SEM) images of NiCo-LDH electrode materials at versatile magnifications (**a**,**b**), NiCo_2_O_4_ electrode materials at different magnifications (**c**,**d**), and NiCo_2_O_4_@NiCo_2_O_4_ electrode materials at versatile magnifications (**e**,**f**).

Morphologies of bare NiCo-layered double hydroxides (LDH) before annealing, plain NiCo_2_O_4_ before electrochemical deposition, and the laminated nanoflakes were observed utilizing SEM. As can be seen from Figure 3a–d, NiCo-layered double hydroxides (LDH; before annealing) and bare NiCo_2_O_4_ nanoflakes covered the surface of the substrate uniformly after the hydrothermal process. The creation of the nanoflake layer was based on non-homogeneous growth and nucleation, because of lower interfacial nucleation energy on the nickel foam [25,26]. The nanoflakes are very thin, causing complete employment of the active material. Figure 3e,f depict the laminated nanoflake thin layer after the electrochemical deposition process. The nanoflakes’ thickness increases and the surface is enclosed with several very thin nanoflakes (leaf-like), creating a very porous laminated structure. The second laminate nanoflakes are interconnected but do not completely cover the first laminate. Voids or pores between nanoplatelets of the first laminate and second laminate act as operative transportation canals for electrolytes throughout charge−discharge processes. Most of the laminated nanoflakes were very accessible to the electrolyte, yielding high specific capacitance. Both the first laminate and the second laminate were ultrathin; therefore, the electrolyte diffused to the bottom of the electrode materials.

A thin film of NiCo_2_O_4_ flakes was created on the surface of each nanocactus-shaped NiCo_2_O_4_ after electrodeposition and annealing, creating a laminated structure. The NiCo_2_O_4_ nanoflakes had a porous structure and were interconnected with one another, which was proven from the SEM image at higher magnification [23,24]. The porous structure helped with electrolyte permeation, and the interconnected nature enabled quick electron and ion transportation.

**Figure 4:**
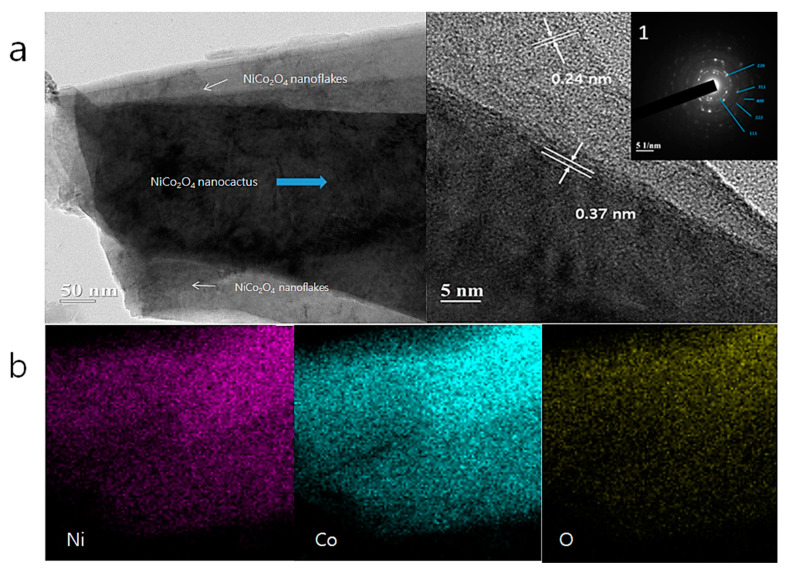
(**a**) TEM image and HRTEM image of individual NiCo_2_O_4_@NiCo_2_O_4_ hierarchical nanostructures and (a1) inset show the selected area electron diffraction (SAED) and (**b**) corresponding EDS mapping results.

Transmission electron microscopy (TEM) measurements were carried out to further investigate the structure of the NiCo_2_O_4_@NiCo_2_O_4_ hierarchical nanostructures. Figure 4a shows the TEM image of isolated NiCo_2_O_4_@NiCo_2_O_4_ hierarchical nanostructures; the NiCo_2_O_4_ nanocactus and NiCo_2_O_4_ nanoflakes can be seen. The SAED pattern (Figure 4a1) shows bright spots, proving the single crystallinity of NiCo_2_O_4_ nanoflakes, while the diffraction rings reveal the development of NiCo_2_O_4_ nanoflakes on the surface of the NiCo_2_O_4_ nanocactus. These results can be indexed to (111), (220), (311), (222), and (400) planes of NiCo_2_O_4_, which also corresponded to the XRD results. High-resolution transmission electron microscopy (HRTEM) was used for further characterization. The lattice spacing of the NiCo_2_O_4_ nanocactus was approximately 0.37 nm. The lattice spacing of the NiCo_2_O_4_ nanoflakes with the shell structure was approximately 0.27 nm, close to the theoretical interplane spacing of spinel NiCo_2_O_4_ (311) planes. The thickness of the inner and outer layers can be seen from the boundary. Thus, the NiCo_2_O_4_ nanoflakes are composed of 1–2 layers of NiCo_2_O_4_ atomic sheets and the NiCo_2_O_4_ nanocactuses are composed of 4–5 layers. Figure 4b presents the EDS mapping images of the Co, Ni, and O elements from the specimen depicted in Figure 4a. This result illustrates the chemical component and spatial distribution in the NiCo_2_O_4_@NiCo_2_O_4_ structure, explaining that the specimen is a hierarchical nanostructure.

**Figure 5:**
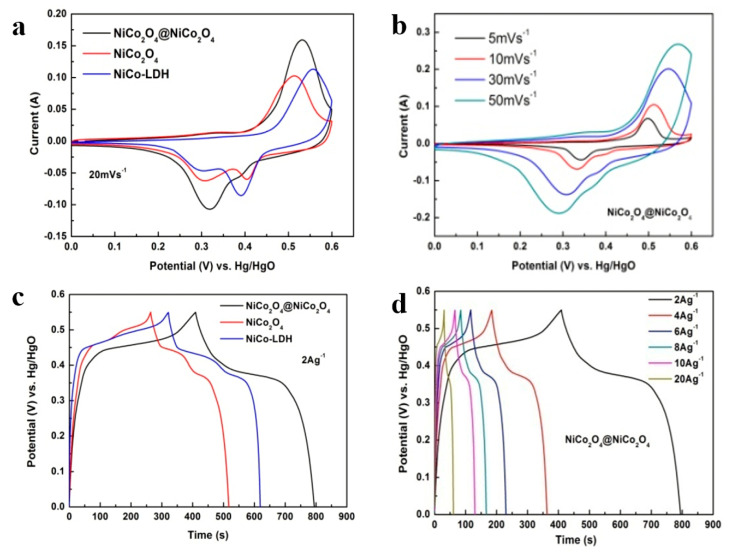
Cyclic voltammetry (CV) plots of (**a**) the NiCo_2_O_4_@NiCo_2_O_4_ nanoflake array, bare NiCo_2_O_4_ nanoflake, and NiCo-LDH samples at 20 mV s^−1^ scanning rate. (**b**) CV plots of the NiCo_2_O_4_@NiCo_2_O_4_ sample at various scan rates. Charge discharge (CD) plots of (**c**) the three samples at 2 A g^−1^ current density and (**d**) NiCo_2_O_4_@NiCo_2_O_4_ at various CDs.

To calculate the specific capacitances and to study the electrochemical characteristics of the electrodes, the cyclic voltammetry (CV) tests were carried out. Figure 5a shows the CV plot at a 20 mV s^−1^ scan rate for the NiCo_2_O_4_@NiCo_2_O_4_ nanoflake, NiCo_2_O_4_ nanoflake, and NiCo-LDH samples. The region integrated within the potential–current graph of the laminated structure of the NiCo_2_O_4_@NiCo_2_O_4_ sample is bigger compared to NiCo_2_O_4_ and NiCo-LDH samples, indicating greater electrochemical reactions of the NiCo_2_O_4_@NiCo_2_O_4_ sample. The NiCo_2_O_4_ with the large surface area and high porosity is accountable for quick electron transfer and increased ion diffusion in the NiCo_2_O_4_@NiCo_2_O_4_ sample [27]. The positions of the redox peaks of the three samples are quite dissimilar, which is attributed to the alteration in behaviors of electrode polarization through CV experiments. The following reactions describe the redox reaction activities in a high pH electrolyte:

(9) $$NiCo_{2}O_{4}+OH^{-}+H_{2}O↔NiOOH+2CoOOH+e^{-}$$

(10) $$CoOOH+OH^{-}↔CoO_{2}+H_{2}O+e^{-}$$

Current voltammetry plots of the NiCo_2_O_4_@NiCo_2_O_4_ sample attained at versatile scan rates are presented in Figure 5b. The curve shapes show typically battery-type capacitive characteristics for the NiCo_2_O_4_@NiCo_2_O_4_ electrode. The redox peaks were broad and weak, initiating from faradaic redox reactions associated with M-O/M-O-OH, where M signifies both ions of Co and Ni [26,28]. The current increased with an enhancement in the scan rate from 5 to 50 mV s^−1^, whereas the CV curve shape remained unaffected, except for changes in peak positions.

To examine the capacitances of the three samples, the galvanostatic charge–discharge (GCD) experiments were carried out in the 0−0.55 V voltage range. The discharge capacitance values of NiCo-LDH, NiCo_2_O_4_@NiCo_2_O_4_ nanoflake arrays, and bare NiCo_2_O_4_ at a discharge capacitance of 2 A g^−1^ are presented in Figure 5c. The bare NiCo_2_O_4_ sample delivered a lower specific capacitance than the laminated nanoflake array electrode. The laminated nanoflake array electrode’s discharge areal capacitance was measured as 1398.73 F g^−1^, which was larger than the values for bare NiCo_2_O_4_ (920.5 F g^−1^) and NiCo-LDH (1082.6 F g^−1^). The specific capacitances of the three samples at different discharge current densities were quantified and are shown in Figure 5d. The NiCo_2_O_4_@NiCo_2_O_4_ nanoflake array samples had specific capacitances of 1398.73, 1296.814, 1239.84, 1201.99, 1171.98, and 1090.9 F g^−1^ at charge discharges (CD) of 2, 4, 6, 8, 10, and 20 A g^−1^, respectively, higher than those of bare NiCo_2_O_4_ arrays (920.5, 887.63, 861.91, 841.06, 821.21, and 758.4 F g^−1^, respectively) and NiCo-LDH (1082.6, 1018.9, 974.39, 939.811, 911.89, and 812.29 F g^−1^, respectively).

**Figure 6:**
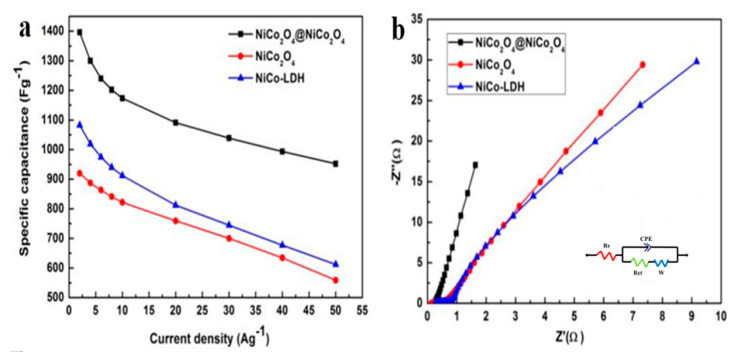
(**a**) Specific capacitances of different samples at different CDs and (**b**) Nyquist curves of different samples.

Figure 6 depicts the current density vs. specific capacitance for different samples. The specific capacitances (examined from the discharge curves) of the NiCo_2_O_4_@NiCo_2_O_4_ sample are 1398.73, 1296.814, 1239.84, 1201.99, 1171.98, 1090.9, 1038.927, 993.236, and 953.18 F g^−1^ at 2, 4, 6, 8, 10, 20, 30, 40, and 50 A g^−1^ CDs, respectively. As can be seen, by enhancing the rate of the charge–discharge from 2 to 50 A g^−1^, the capacitance retention of the hybrid NiCo_2_O_4_@NiCo_2_O_4_ electrode is increased to approximately 68.146%. The hybrid sample retained a high capacitance of 953.18 F g^−1^, even at a high CD of 50 A g^−1^, specifying the huge benefits of this exclusive complex structure [29,30]. Figure 6b shows EIS test graphs identifying the ion diffusion and electron transfer in the three samples. As can be seen, the two impedance spectra are identical; each contains linear and semicircle shapes at low and high frequencies, respectively [31]. The summary of the contact resistance at the active current–material collector interface, intrinsic resistances of the active materials, and electrolyte ionic resistance is called the internal resistance (Rs), which is attained from the intercept of the plot on the real axis. The Nyquist plot semicircle relates to faradaic reactions and its diameter signifies the resistance of interfacial charge transfer. In the low-frequency area, the NiCo_2_O_4_@NiCo_2_O_4_ sample shows a shorter line and higher slope, signifying smaller variation in diffusion paths and a quicker OH^–^ diffusion rate. The findings imply that the mixture of fast low electron transfer resistance and ion diffusion increases the electrochemical activities of the sample with the laminated structure [26,32]. The self-supporting nature of the sample, as well as the developed structures, permits quick ion–electron transport devoid of polymer binders and conductive additives, which usually increase the interfacial resistance [33].

**Figure 7:**
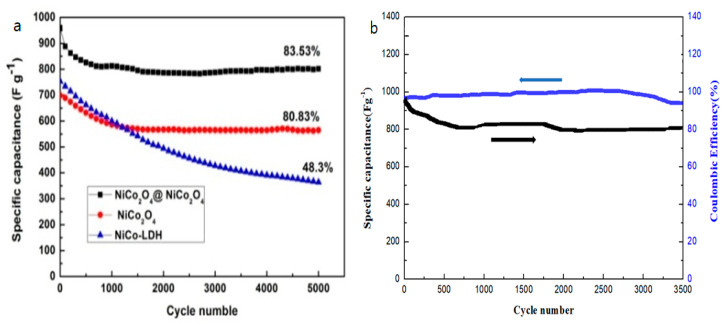
(**a**) Cycling stability test of different samples at 20 A g^−1^ CD for 5000 cycles and (**b**) the NiCo_2_O_4_@NiCo_2_O_4_ electrode’s cycling stability and Coulombic efficiency at a CD of 20 A g^−1^ for 3500 cycles.

High cycling stability is an indicator of the supercapacitor activity enhancement of the NiCo_2_O_4_@NiCo_2_O_4_ nanoflake sample. Figure 7a displays the cyclability of different samples at a CD of 20 A g^−1^ and a potential range of 0 and 0.55 V over 5000 cycles. As can be seen, the specific capacitances of three samples declined slowly in the first 1000 cycles, which may have been attributed to the fall-off of the active material. The cycling stability increased in the laminated nanoflake array sample, for which the specific capacitance was 804 F g^−1^ after 5000 cycles; it retained 83.53% of the highest value, greater than that of bare NiCo_2_O_4_ (556.18 F g^−1^ with 80.83%) and NiCo-LDH (381.751 F g^−1^ with 48.3%). The developed NiCo_2_O_4_@NiCo_2_O_4_ electrode, therefore, shows outstanding and significant electrochemical stability for a long cycle life at high CD [33.34]. As shown in Figure 7b, at low current density some side reactions occurred in the electrochemical redox reaction, leading to incomplete discharge. When the current density amplified, the charge and discharge time declined. The electrochemical process was primarily affected by the electric double layer, so the Coulomb efficiency became larger.

Various parameters contributed to the significant cycling stability and great specific capacitance of the NiCo_2_O_4_@NiCo_2_O_4_ sample. Compared with other similar works, the electrodeposition of the second laminate NiCo_2_O_4_ nanoflake by CV scan method confirmed the uniform distribution of the second laminate NiCo_2_O_4_ nanoflake. The cathodic reduction (NO_3_^−^ + H_2_O + 2e^−^→NO_2_ + 2OH^−^) and uniform deposition of Ni-Co LDH on the working electrode were controlled by current fluctuation. Meanwhile, the second laminate NiCo_2_O_4_ nanoflake did not completely enclose the first laminate NiCo_2_O_4_ and was interconnected. Most of the laminated nanoflakes were highly reachable by the electrolyte for energy storage, resulting in significant specific capacitance. Both the first and the second laminates were ultrathin in a complex and porous structure; hence, the electrolyte diffused to the bottom of the deposited sample, which led to the participation of all active materials in the process of electrochemical charge storage, resulting in greater specific capacitance. Then, the electrolyte infiltrated the complex porous structure of the electrode, as the large open canals amid the nanoflakes allowed easier permeation of the electrolyte to the inner area of the sample, enhancing the active material usage. Second, the hydrothermally developed NiCo_2_O_4_ nanoflakes were covered by NiCo_2_O_4_ nanoflakes through electrochemical deposition. They retained the structural reliability of the first laminate and caused easy electron transport for the faradaic reaction during the charge−discharge practice. Finally, the NiCo_2_O_4_ nanoflakes that were linked with one another and supported each other made a network, which led to structural stabilization. The NiCo_2_O_4_@NiCo_2_O_4_ electrode maintained the retention ratio of 83.53%, measured under the current density of 20 A g^−1^ (60 mA cm^−2^), which indicated our electrode can work at a higher current density and effectively prevent the aggregation of adjacent nanoflakes during the charge–discharge process.

**Figure 8:**
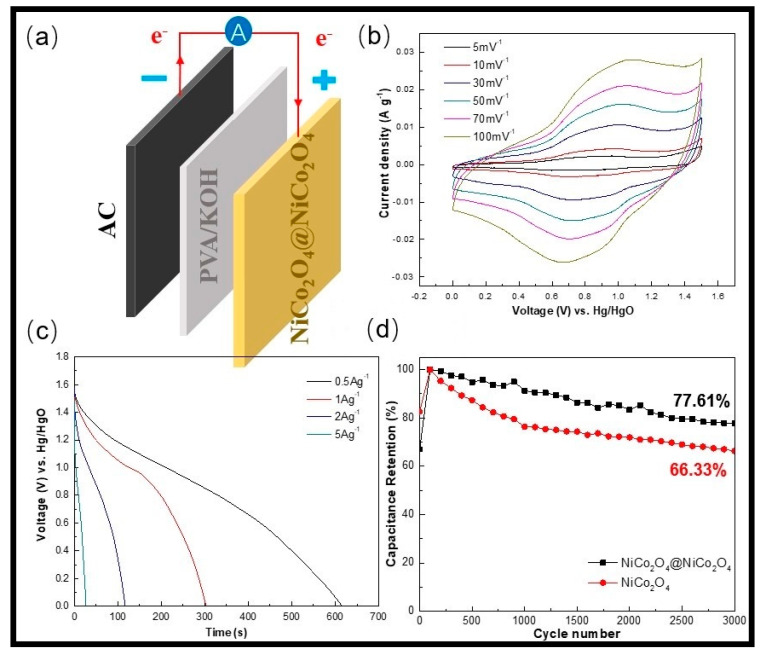
(**a**) Graphical presentation of the asymmetric supercapacitor (ASC) device, (**b**) CV curves of the ASC device at versatile scan rates, (**c**) charge–discharge curves at versatile current densities, and (**d**) the cycle stability of the NiCo_2_O_4_ and NiCo_2_O_4_@NiCo_2_O_4_ devices at 10 Ag^−1^ for 3000 cycles.

An asymmetric supercapacitor (ASC) device was fabricated to prove the great capacitive performance of the NiCo_2_O_4_@NiCo_2_O_4_ on the Ni substrate. NiCo_2_O_4_@NiCo_2_O_4_@ nickel foam (NF) was selected as a positive electrode and AC@NF was selected a negative electrode. The presynthesized KOH/PVA substance electrolyte was added amid negative and positive electrodes as a separator (see Figure 8a). Figure 8b shows the CV curves of the ASC at various scan rates from 0 to 1.5V. As can be seen, the shape of the CV curves did not change with the change in scan rate from 5 mV^−1^ to 100 mV^−1^_,_ thus suggesting the device has good and quick charge–discharge properties.

The GCD curves of the asymmetric supercapacitor at different CDs are presented in Figure 8c. Based on the discharge curves, the following formulas were used to calculate the values for the power density (*P*) and energy density (*E*):

(11) $$P=\frac{E}{\Delta t}$$

(12) $$E=\frac{1}{2}CV^{2}$$

Here, *V* (V) and *E* (Wh kg^−1^) are the voltage range, respectively, excluding the IR drop (ohmic potential loss) and the energy density; *Δt* and *P* (W kg^−1^) are the discharge time and the average power density, respectively.

Based on the specific capacity values, the energy density values of the NiCo_2_O_4_@NiCo_2_O_4_//AC were 46.46, 45.15, 41.47, and 31.54 Wh kg^−1^ at power densities of 269.77, 540.09, 996.94, and 2703.4 W kg^−1^, respectively.

The device’s cycle stability for 3000 cycles at 10 Ag^−1^ is presented in Figure 8d. As can be observed from the plot, the specific capacity increased initially, which was attributed to the active material activation during the initial cycles. As the electrolyte penetrates the active materials, more hybrid nanostructures are activated after the intercalation and deintercalation process, resulting in enhancement of the capacitance [32]. For the NiCo_2_O_4_@NiCo_2_O_4_ electrode, the capacitance retention values at 1000 and 3000 cycles were around 92% and 77.61%, respectively. In the meantime, the pure NiCo_2_O_4_ electrode’s capacitance values at 1000 and 3000 cycles remained at 75% and 66.33%, respectively. The findings showed that the composite electrodes exhibited outstanding performance regarding cycle stability with higher CD [33].

The outstanding electrochemical properties of the NiCo_2_O_4_@NiCo_2_O_4_ electrodes were the result of the subsequent conditions: (1) the NiCo_2_O_4_@NiCo_2_O_4_ nanoflake was directly grown on Ni foam without any binders, which significantly improved the connected conductivity between active materials and the substructure, as well as facilitated the electron transport; (2) the unique hierarchical nanostructure with the large surface area could expose more active sites for redox reactions and had higher specific capacitance; (3) the leaf-like morphology of the active materials provided opening space between adjacent nanoflakes, maintaining the structural integrity during long-term charge–discharge processes. We et al. evaluated the specific capacitance of nickel cobaltite nanograss/CNT synthesized on stainless steel wire mesh at current densities of 1 and 50 A g^−1^. The specific capacitances of the electrode for different current densities (1 and 50 A g^−1^) were 1223 and 1070 F g^−1^, respectively [32]. In another study performed by Liu et al., the electrochemical performance of NiCo_2_O_4_@ NiCo_2_O_4_ nanoflake arrays was examined. The specific capacitance of the composite reached 2.20 F cm^−2^ at a current density of 5 mA cm^−2^. They found that the specific capacitance of the composite was around 2.17 F cm^−2^ after 4000 cycles [34].

## 4. Conclusions

In this work, a binder-free, porous NiCo_2_O_4_@NiCo_2_O_4_ with laminated nanoflakes was synthesized on NF by combined hydrothermal and cyclic voltammetry deposition techniques for supercapacitor applications. Electrochemical experiments were carried out to evaluate the performance of the material for supercapacitors. The results indicated the NiCo_2_O_4_@NiCo_2_O_4_ material maintained good electrochemical stability at high CD. The proposed cyclic voltammetry deposition can be extended to other core–shell metal oxides to improve their capacitive performance for energy storage.
